# Apathy in Dementia: A Pilot Study Providing Insights from Neuropsychiatric and Radiological Perspectives

**DOI:** 10.3390/jcm14061822

**Published:** 2025-03-08

**Authors:** Ozlem Totuk, Sevki Sahin

**Affiliations:** Department of Neurology, Hamidiye Faculty of Medicine, Sancaktepe Sehit Prof. Dr. Ilhan Varank SUAM, University of Health Sciences, Istanbul 34785, Turkey

**Keywords:** dementia, apathy, neuropsychiatric symptoms, cognitive functions, radiological imaging

## Abstract

**Background:** Apathy is a common neuropsychiatric symptom in all stages of dementia, significantly complicating patient management. This study examines the prevalence of apathy across Alzheimer’s Disease (AD), Lewy Body Dementia (LBD), Frontotemporal Dementia (FTD), and Vascular Dementia (VD) and explores its associations with cognitive functions, neuropsychiatric symptoms, and magnetic resonance imaging (MRI) findings. **Methods:** This retrospective, cross-sectional study included 200 patients diagnosed with AD, LBD, FTD, and VD along with 100 healthy controls (HCs). Apathy was assessed using the Apathy Evaluation Scale. Depression and anxiety in patients were evaluated using the Geriatric Depression Scale and the Geriatric Anxiety Scale, respectively. Cognitive function was measured with the Mini-Mental State Examination (MMSE) and Addenbrooke’s Cognitive Examination-Revised (ACE-R). MRI findings were evaluated using atrophy scales that are routinely utilized in dementia assessments. **Results:** Apathy was significantly more prevalent in dementia and MCI patients compared to HC. However, there were no significant differences in apathy prevalence among dementia subtypes. Apathy showed no significant correlation with depression, anxiety, or cognitive performance. Notably, MRI analysis revealed a strong association between apathy and orbitofrontal (OF) sulci atrophy. **Conclusions:** Apathy is a critical symptom in dementia, linked to OF atrophy and presenting challenges in management. These findings emphasize the importance of integrating apathy assessments in clinical practice. Larger, longitudinal studies are needed to further clarify the pathophysiology and management of apathy in dementia.

## 1. Introduction

Dementia is characterized by the progressive deterioration of multiple cognitive functions, leading to functional impairment. Alongside these cognitive deficits, neuropsychiatric (NP) symptoms are also commonly observed [1]. While traditionally believed to manifest only in the advanced stages of the disease, recent studies have shown that NP symptoms can emerge as early as the prodromal stages. These symptoms significantly contribute to the burden experienced by caregivers [2,3].

Research on Alzheimer’s Disease (AD), in particular, has demonstrated that depression is more prominent in the early stages of the disease, whereas delusions and hallucinations tend to dominate in later stages. Apathy, however, is frequently observed throughout all stages of AD [4]. Notably, apathy is not limited to AD but is also present across various types of dementia [5].

Apathy is characterized by symptoms such as a loss of motivation, reduced goal-directed behavior, diminished cognitive activity, and decreased emotional expression, persisting for at least four weeks [6]. It is among the most common neuropsychiatric symptoms in dementia syndromes and has been suggested to play a role in the quality of life in patients and caregivers. Also, if not specifically assessed, apathy can be difficult to recognize and challenging to manage [4,7].

The underlying mechanism of apathy remains unclear. Orexin, a neuropeptide, regulates various neurological processes, including wakefulness, motor function, appetite, stress response, and reward processing. The dysregulation of the orexin system has been associated with symptoms such as agitation, anxiety, sleep disturbances, and apathy in dementia and parkinsonian syndromes. However, it remains uncertain whether these alterations in the orexin system contribute to neurodegeneration or occur as a consequence of the disease. Given its potential role in these disorders, pharmacological strategies targeting orexin receptors may offer therapeutic benefits, warranting further investigation [8].

Apathy is broadly linked to dysfunction within the medial frontal cortex, particularly the anterior cingulate cortex (ACC) and orbitofrontal cortex (OFC), as well as subcortical structures such as the ventral striatum (VS), medial thalamus, and ventral tegmental area (VTA), or the disrupted connectivity among these regions [9]. In conditions where volumetric analyses or functional MRI assessments of these structures are challenging, routine and simple MRI evaluation measures may serve as a useful alternative.

Studies indicate that apathy is a significant feature of dementia and may even serve as a prognostic marker [9]. Thus, in this study, we investigated the frequency of apathy in dementia patients across different types of dementia and examined its relationship with other neuropsychiatric symptoms, cognitive functions, and cranial imaging findings.

## 2. Materials and Methods

### 2.1. Participants

This study included 200 patients followed at the Dementia Clinic of Sancaktepe Prof. Dr. İlhan Varank Training and Research Hospital, along with 100 healthy volunteers (HVs). The diagnosis of AD was made based on the criteria of the National Institute on Aging and the Alzheimer’s Association (NIA-AA) [10]. For Frontotemporal Dementia (FTD), the International Behavioural Variant FTD Criteria Consortium criteria were applied [11]. The International Society of Vascular Behavioural and Cognitive Disorders criteria were used for Vascular Dementia (VD) [12], while the revised criteria proposed by McKeith et al. in 2017 were employed for Lewy Body Dementia (LBD) [13].

Patients were included in the study regardless of dementia stage. All participants were informed about the study, and consent was obtained either from the patients themselves or their legal guardians. Participants were matched for age, sex, and education level. Patients with any condition impairing physical functionality within the past six months were excluded. Also, individuals with diagnosed psychiatric disorders, uncontrolled metabolic or infectious conditions, or sensory impairments such as hearing or vision loss that could compromise test adherence were excluded from the study.

Demographic data and disease onset durations were recorded. Apathy was evaluated in both the patient and HV groups using the Apathy Evaluation Scale. In the patient group, depression and anxiety levels were assessed using the Geriatric Depression Scale and the Geriatric Anxiety Scale. Cognitive status was evaluated with the Mini-Mental State Examination (MMSE) and Addenbrooke’s Cognitive Examination-Revised (ACE-R). Magnetic resonance imaging (MRI) findings were analyzed using atrophy scales.

### 2.2. Data Collection Tools

#### 2.2.1. Mini-Mental State Examination (MMSE)

The MMSE, developed by Folstein et al. in 1975, has undergone validation studies in Turkey [14,15]. It evaluates cognitive function across five sections: orientation, registration, attention/calculation, language, and recall. The maximum score is 30. Score ranges are categorized as follows: a range of 0–9 indicates severe cognitive impairment; a range of 10–19 indicates moderate cognitive impairment; a range of 20–23 indicates mild cognitive impairment, and 24–30 is considered “normal cognitive functioning”.

#### 2.2.2. Addenbrooke’s Cognitive Examination-Revised (ACE-R)

The ACE-R is particularly sensitive in differentiating early-stage dementia. It assesses five subdomains: attention/orientation, memory, verbal fluency, language, and visuospatial abilities. The test has been adapted and validated for use in a Turkish context [16,17].

#### 2.2.3. Apathy Evaluation Scale (AES)

The AES measures the prevalence and severity of apathy by evaluating behavioral, cognitive, and social/emotional domains of indifference and a lack of motivation over the past four weeks. A Turkish validity and reliability study has been conducted [18]. The scale comprises 18 items, with scores ranging from 18 to 72, where higher scores indicate greater levels of apathy. Both self-report and clinician-administered forms are available; in this study, the self-report form was used. Scores of 25 or above were considered indicative of apathy.

#### 2.2.4. Geriatric Depression Scale (GDS)

In our study, GDS was preferred as the depression inventory due to its easier applicability and better comprehension in the elderly population. The GDS is a self-report scale consisting of 30 questions designed to detect depressive symptoms in the elderly population. The scale has been validated for reliability and validity in the Turkish population [19]. Each question uses a binary response format (“yes” or “no”), with responses indicative of depression scored as 1 point and others scored as 0. Higher scores indicate greater levels of depressive mood. Scoring is categorized as follows: 0–4 points: normal, 5–8 points: mild depressive symptoms, 9–11 points: moderate depressive symptoms, and ≥12 points: severe depressive symptoms.

#### 2.2.5. Geriatric Anxiety Scale (GAS)

The GAS is a self-report tool designed to assess anxiety symptoms in the elderly population. It evaluates three subdomains: somatic, cognitive, and emotional. The Turkish version consists of 28 items, with the first 23 items contributing to the total score [20]. Each item is scored on a four-point Likert scale: “Never (0)”, “Sometimes (1)”, “Often (2)”, and “Always (3)”. Items 24–28 are used by clinicians to determine the domain of anxiety but are not included in the total score. Participants are asked to reflect on their symptoms over the past week. The total score ranges from 0 to 75, with higher scores indicating higher levels of anxiety.

### 2.3. Radiological Imaging Findings

All scans were performed on 1.5 T MRI units (GE Signa Explorer; GE, Milwaukee, WI, USA). Non-contrast T1-weighted, T2-weighted, and FLAIR sequences were obtained. The imaging parameters were as follows: axial and sagittal T1-weighted MRI (TR/TE: 543/24 ms), axial and coronal T2-weighted MRI (TR/TE: 5724/102 ms), and axial FLAIR images (TR/TE: 8000/86 ms). The neurology expert (SND) performed all assessments and was blinded to patient demographic and clinical data.

Koedam, medial temporal atrophy (MTA), orbitofrontal cortex grading, and Fazekas scores were evaluated by a single neuroradiologist (NHS) with over five years of clinical experience.

### 2.4. Fazekas Grading System

Vascular lesions were analyzed in terms of their location (juxtacortical, periventricular, and deep brain structures), subcortical atrophy, and lesion burden using the Fazekas grading system [21] (Box 1).

Box 1 **Fazekas grading system.**

**Fazekas 0:** No lesions or a single punctate lesion (white matter hyperintensity).

**Fazekas 1:** Multiple punctate lesions.

**Fazekas 2:** Beginning to coalesce lesions (bridging).

**Fazekas 3:** Large, confluent lesions.

Subcortical atrophy was calculated using the ventricle-to-brain ratio, without differentiating white and gray matter. Lesions in white matter adjacent to the cortex were classified as juxtacortical, while signal abnormalities extending up to 5 mm from the ventricles to the cortex were categorized as periventricular.

### 2.5. Medial Temporal Atrophy (MTA) Score

The medial temporal atrophy (MTA) score was qualitatively evaluated based on the degree of atrophy in the choroid fissure, temporal horn, and hippocampal volume, following the criteria described by Frisoni et al. [22,23] (Box 2).

Box 2 **Medial temporal atrophy (MTA) scoring system.**

**MTA 0:** Normal choroid fissure, temporal horn, and hippocampal volume.

**MTA 1:** Mildly enlarged choroid fissure.

**MTA 2:** Moderately enlarged choroid fissure, with mild enlargement of the temporal horn and a mild reduction in hippocampal volume.

**MTA 3:** Significantly enlarged choroid fissure, with moderate enlargement of the temporal horn and a moderate reduction in hippocampal volume.

**MTA 4:** Severely enlarged choroid fissure, significant enlargement of the temporal horn, and a marked reduction in hippocampal volume.

### 2.6. Koedam Score

Parietal atrophy was qualitatively assessed using the Koedam score, based on the degree of atrophy in the parietal region in the sagittal plane, as described by Koedam et al. [24,25] (Box 3).

Box 3 **Koedam (posterior/parietal) atrophy score.**

**Koedam 0:** No sulcal widening or precuneus atrophy.

**Koedam 1:** Mild sulcal widening and mild precuneus atrophy.

**Koedam 2:** Marked sulcal widening and moderate parietal cortical atrophy.

**Koedam 3:** Severe parietal atrophy with a “knife-edge” sulcal appearance.

### 2.7. Orbitofrontal Sulci Grading

The orbitofrontal (OF) sulci atrophy grading system is a visual rating scale designed to assess the degree of cortical atrophy in the orbitofrontal region by evaluating the widening of the sulci on MRI scans. This qualitative tool has been particularly useful in differentiating the atrophy pattern between FTD and AD. The assessment is usually performed on T1-weighted coronal or axial sequences of MRI where experienced raters visually inspect the OF region [26] (Box 4).

Box 4 **The orbitofrontal (OF) sulci atrophy grading system.**

**Grade 0:** No atrophy is evident; the orbitofrontal sulci appear normal.

**Grade 1:** Mild atrophy, characterized by the subtle widening of the sulci and minimal cortical thinning.

**Grade 2:** Moderate atrophy, with slightly noticeable widening of the sulci and evident cortical thinning.

**Grade 3:** Moderate atrophy, with more noticeable widening of the sulci and evident cortical thinning.

**Grade 4:** Severe atrophy, featuring marked widening of the sulci accompanied by significant cortical thinning.

### 2.8. Statistical Analysis

Frequency analysis was conducted to examine the demographic characteristics of the study participants. Subsequently, scores for the subdimensions and total dimensions of the measurement tools were calculated, and descriptive statistics for these scores were presented. Average comparison tests were performed to analyze the subdimensional and overall scores of the scales in relation to specific participant characteristics.

Before performing comparisons, the skewness and kurtosis values of the variables were checked to ensure that they fell between −3 and +3, confirming the normality of the distribution. Descriptive statistics for numerical variables were reported as mean (Mean), standard deviation (SD), median (Med.), minimum (Min.), and maximum (Max.) values.

A statistical significance level of *p* < 0.05 was considered in all analyses. Statistical calculations were performed using SPSS version 27.

## 3. Results

The frequency analysis results for the descriptive characteristics of the participants are summarized as follows. A total of 200 dementia patients (110 women) and 100 healthy volunteers (HVs) (60 women) participated in the study. The mean age of the patient group was 71.31 ± 11.71 years, while the mean age of the HV group was 68 ± 3.5 years.

In terms of education levels, 146 participants (48.67%) had 0–5 years of education; a total of 105 participants (35%) had 6–11 years of education, and 49 participants (16.33%) had 12 or more years of education. The years of education of healthy volunteers and patients were selected to be similar to each other.

The distribution of participants by diagnosis showed that 124 patients were diagnosed with AD (62%), 30 patients with VD (15%), 26 patients with FTD (13%), and 20 patients with LBD (10%).

In terms of disease duration, 135 patients (67.5%) had a disease duration of 1–3 years; a total of 38 patients (19%) had a duration of 3–5 years, and 27 patients (13.5%) had a disease duration of 5 years or more.

In this study, the mean, standard deviation, and range of measurement tools are presented in Table 1.

The prevalence of apathy in the patient group was calculated as 88.24%.

**Figure 1:**
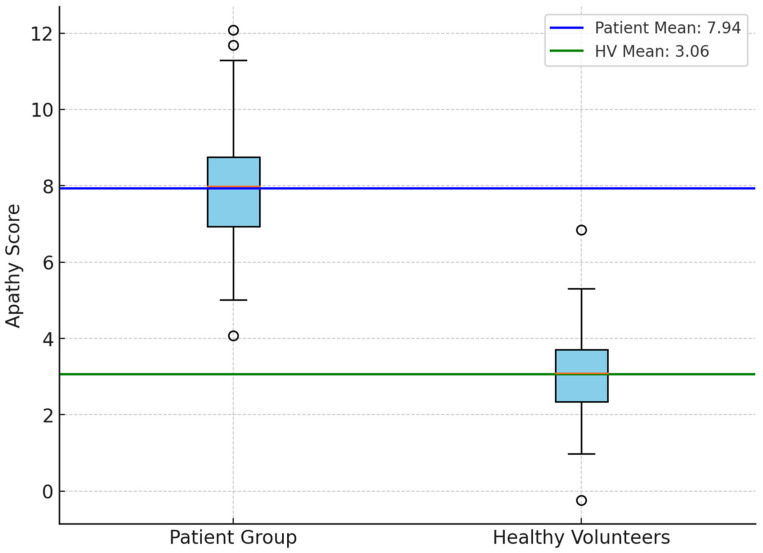
Comparison of apathy scores between patients and healthy volunteers (HVs).

An independent sample *t*-test was performed to compare the apathy evaluation scores between the patient group and the healthy volunteer (HV) group. The results revealed a statistically significant difference, with the patient group exhibiting higher apathy scores compared to the HV group (t = 9.69, *p* < 0.001; *p*-value: 1.54 × 10^−17^) (Figure 1). When the prevalence of apathy was analyzed by diagnosis, the rates were found to be 100.0% in LBD, 85.71% in AD, 86.67% in VD, and 84.62% in FTD.

**Figure 2:**
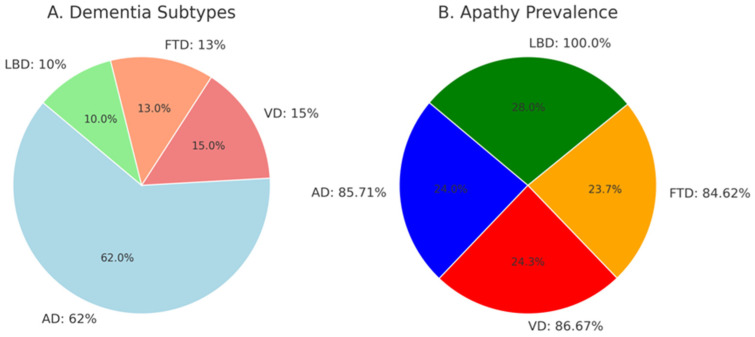
(**A**) Distribution of dementia subtypes in the study group. (**B**) Apathy prevalence rates across those groups. AD: Alzheimer’s Dementia, FTD: Frontotemporal Dementia, and LBD: Lewy Body dementia.

The Chi-square test, conducted to compare the prevalence of apathy among diagnostic groups, revealed no statistically significant difference (χ^2^ = 2.21, *p* = 0.697) (Figure 2). Similarly, Chi-square and *t*-test analyses demonstrated no statistically significant relationships between the presence of apathy and gender (*p* = 0.617), age (*p* = 0.463), education duration (*p* = 0.716), or disease onset year (*p* = 0.067).

**Figure 3:**
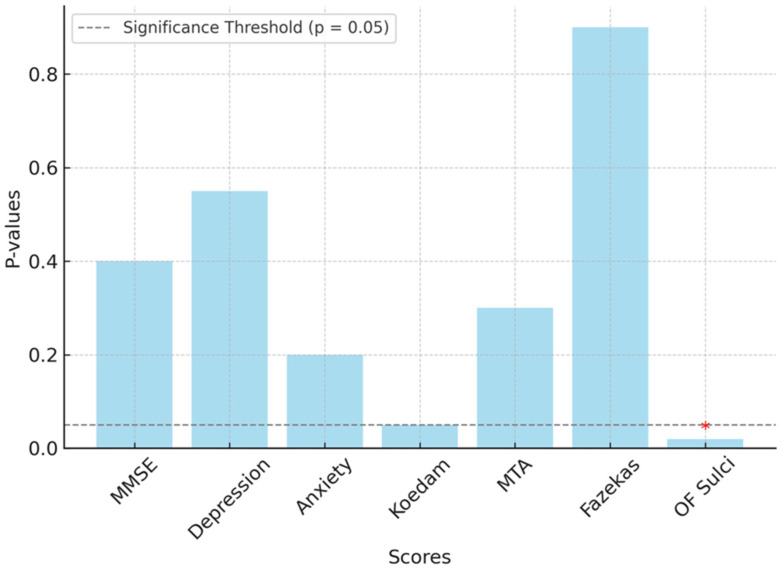
*p*-Value comparison for various variables related to apathy (MMSE: Mini-Mental State Examination; OF: orbitofrontal; *: Indicates a statistically significant value).

Additionally, comparison tests showed no statistically significant associations between the presence of apathy and cognitive status, as assessed using Addenbrooke’s Cognitive Examination-Revised (ACE-R) and the Mini-Mental State Examination (MMSE) (*p* = 0.505 and *p* = 0.443, respectively). No significant relationships were observed between apathy and depression (*p* = 0.557) or anxiety (*p* = 0.228) (Figure 3).

The analysis examining the relationship between the presence of apathy and MRI atrophy scales found no significant differences for the Koedam scale (t = −1.94, *p* = 0.073), MTA scale (t = −1.04, *p* = 0.318), or Fazekas scale (t = −0.10, *p* = 0.921). However, a significant difference was observed between the apathetic and non-apathetic groups on the OF sulci atrophy grade (t = −2.18, *p* = 0.048) (Figure 3). However, no lateralizing difference was detected between the right and left hemispheres.

## 4. Discussion

This study investigated the frequency of apathy in dementia patients, differences in the presence of apathy among various types of dementia, its relationship with cognitive function and other neuropsychiatric symptoms, and its association with MRI findings.

The prevalence of apathy in dementia varies widely in the literature. A review reported prevalence ranges of 26–82% for AD, 28.6–91.7% for VaD, and 54.8–88.0% for FTD [27]. In some studies, the overall prevalence across all dementia types has been reported as 36% [28]. In the present study, the frequency of apathy in the patient group was calculated as 88.24%. When analyzed by specific diagnoses, the prevalence of apathy was 100.0% in LBD, 85.71% in AD, 86.67% in VD, and 84.62% in FTD. Variations in prevalence rates reported in the literature may stem from differences in the tools and criteria used to assess apathy. In addition to other neurodegenerative dementias, the VD group in our study was carefully composed of cases that were as free as possible from comorbidities such as uncontrolled metabolic conditions and hypertension. The inclusion of the VD group served as a valuable approach to test the hypothesis that apathy might be more frequently observed in these patients due to its association with cerebrovascular diseases. However, contrary to this hypothesis, our findings revealed a similar prevalence of apathy in the VD group compared to other dementia types, contributing this comparative result to the literature [27].

In studies comparing AD and FTD, apathy has generally been observed more frequently in FTD [29,30]. However, contrary to our hypothesis, no significant differences in the frequency of apathy were found among the dementia types in this study.

FTD can present with distinct behavioral profiles and atrophy patterns. Among its subtypes, the behavioral variant, which predominantly affects the frontal regions [31], is relatively easier to diagnose due to its prominent behavioral manifestations and, consequently, is more frequently identified. To ensure homogeneity in our study, only patients with the behavioral variant of FTD were included. The early diagnosis of FTD could pave the way for promising advancements, especially as new disease-modifying therapies are being developed [31]. For this reason, the validation of the OF sulci atrophy grading system for detecting apathy in the early stages of FTD would provide valuable information.

Although long-term follow-up studies suggest that apathy may increase over time, no significant differences were observed in our study regarding the years since the onset of dementia [32].

The relationship between apathy and depression is frequently discussed in the literature, with publications often focusing on their coexistence or their predictive value for dementia. While some studies suggest an overlap between apathy and depression, these are distinct symptoms. In our study, no significant relationship was found between apathy and depression [33].

In our study, no statistically significant difference was found between the level of cognitive decline and the prevalence of apathy. However, an increase in the frequency of apathy is generally expected with cognitive decline [34]. This finding may be attributed to the similarity of cognitive assessment scores among patients in our study group.

Imaging studies have consistently shown the strongest association with apathy in the OF cortex [35]. In our study, among the atrophy regions evaluated, a significant relationship was found only with the ‘OF sulci’, which reflects OF cortex atrophy without cerebral lateralization. This finding supports the medical literature regarding the pathophysiology of apathy. This easily assessable scale may serve as a potential marker for apathy evaluation in the future [9]. OF sulci atrophy is particularly important in settings where access to functional imaging devices is limited, as structural MRI may provide supportive evidence for the presence of apathy.

Some studies have suggested that white matter changes are associated with apathy and may serve as a predictor of dementia, particularly in cases involving small vessel disease [36,37]. However, in our study, no significant association was observed between apathy and the Fazekas classification, which assesses white matter changes.

### 4.1. Strengths of the Study

This study encompasses multiple types of dementia, which is a significant strength as it allows for comparisons of apathy and other neuropsychiatric symptoms across dementia subtypes, thereby contributing to the existing body of knowledge. Such comprehensive analyses help address gaps in the literature.

In addition to apathy, this study evaluated other neuropsychiatric symptoms, including depression and anxiety. This provides a more detailed understanding of the neuropsychiatric profiles of dementia patients and paves the way for more targeted interventions aimed at improving patient management.

Another notable strength of this study is its investigation of the relationships between apathy, neuropsychiatric symptoms, cognitive functions, and cranial imaging findings. The use of structural MRI, a widely accessible imaging modality, enhances the practical applicability of this study’s findings. OF sulci atrophy was found as the most related finding with apathy for all dementia types in our study.

Finally, the exploration of connections between cognitive assessments and neurological structures offers valuable insights into the neurobiological basis of dementia, thereby advancing our understanding of the disease.

### 4.2. Limitations of the Study

This study was conducted at a single hospital’s dementia clinic, which may limit the generalizability of its findings. Additionally, the retrospective and cross-sectional design represents another limitation. Longitudinal follow-up studies are needed to provide more comprehensive insights into the mechanisms underlying the occurrence of apathy in dementia. Another limitation of our study is that apathy was evaluated without considering the medications used by the patients. However, we ensured that our patients were not subjected to polypharmacy or psychopolypharmacy. Therefore, we believe that we minimized the potential influence of polypharmacy-induced behavioral disturbances [38].

## 5. Conclusions

Apathy is a highly prevalent symptom in dementia, including in its prodromal stages. This study contributes significantly to the literature by demonstrating a relationship between apathy and OF atrophy. The absence of differences in apathy prevalence among dementia subtypes highlights the need for further research into the neurobiological origins of this symptom. Apathy may serve as an important marker for facilitating the early diagnosis of dementia.

However, the retrospective design and single-center data limit the generalizability of the findings. To validate these results, multicenter, longitudinal studies are recommended.

### Recommendations for Future Studies

To better understand the effects of apathy on the dementia process and its long-term impact on prognosis, longitudinal studies are recommended. Such studies could clarify the role of apathy in transitions between different stages of dementia. Incorporating biomarker evaluations would provide deeper insights into the underlying biological mechanisms.

Conducting a multicenter study with a larger and more diverse population would improve the generalizability of the findings. This approach would offer more comprehensive data on the prevalence of apathy and its relationship with dementia subtypes, contributing to a broader understanding of its universal applicability.

## Figures and Tables

**Table 1:** Mean, standard deviation (SD), and minimum and maximum values of measurement tools (ACE-R: Addenbrooke’s Cognitive Examination-Revised; MMSE: Mini-Mental State Examination).

Measurement Tool	Mean ± SD	Minimum	Maximum
**ACE-R point**	53.33 ± 15.24	14.0	97.0
**MMSE point**	20.5 ± 12.35	3.0	72.0
**Geriatric Depression Scale Point**	10.74 ± 8.46	0.0	36.0
**Anxiety Scale Point**	11.36 ± 10.31	0.0	57.0
**Apathy Assessment Scale point**	45.05 ± 15.02	12.0	72.0
**Koedam Score**	1.73 ± 0.94	0.0	3.0
**Medial Temporal Atrophy Score**	1.81 ± 1.03	0.0	4.0
**Fazekas Grade**	1.47 ± 0.98	0.0	3.0
**Orbitofrontal Sulci Atrophy Grade**	0.56 ± 0.75	0.0	2.0

## Data Availability

The original contributions presented in this study are included in the article. Further inquiries can be directed to the corresponding author.
